# Achieving NIR Light-Mediated Tumor-Specific Fenton Reaction-Assisted Oncotherapy by Using Magnetic Nanoclusters

**DOI:** 10.3389/fonc.2021.777295

**Published:** 2021-10-25

**Authors:** Shaoyou Qin, Jinru Xue, Erna Jia, Na Ren, Yongqiang Dong, Changyu Zhou

**Affiliations:** ^1^ Department of Gastroenterology and Hepatology, China-Japan Union Hospital of Jilin University, Jilin University, Changchun, China; ^2^ Department of Thoracic Surgery, China-Japan Union Hospital of Jilin University, Jilin University, Changchun, China; ^3^ Department of Thyroid Surgery, First Affiliated Hospital of Zhengzhou University, Zhengzhou, China

**Keywords:** magnetic nanomaterials, photothermal effect, Fenton chemistry, oncotherapy, long-term toxicity

## Abstract

As an emerging strategy for oncotherapy, Fenton chemistry can efficiently improve the conversion from endogenous H_2_O_2_ into highly toxic ·OH in the whole high-performance therapeutic process. Although promising, the efficiency of Fenton reaction in tumor regions is highly limited by the inefficient delivery of Fenton reagents and the restrictive conditions of tumor microenvironment. One promising strategy against the above limitations is to specifically increase the temperature around the tumor regions. In this study, a novel NIR light-mediated tumor-specific nanoplatform based on magnetic iron oxide nanoclusters (MNCs) was rationally designed and well developed for photothermally enhanced Fenton reaction-assisted oncotherapy. MNCs could accumulate into the tumor regions with the help of an external magnet field to enable T_2_-weighted magnetic resonance (MR) imaging of tumors and MR imaging-guided combined antitumor therapy. Our well-prepared MNCs also revealed excellent photothermal effect upon a NIR light irradiation, promising their further important role as a photothermal therapy (PTT) agent. More importantly, heat induced by the PTT of MNCs could accelerate the release of Fe from MNCs and enhance the efficiency of Fenton reaction under H_2_O_2_-enriched acidic tumor microenvironment. Results based on long-term toxicity investigations demonstrated the overall safety of MNCs after intravenous injection. This work therefore introduced a novel nanoplatform based on MNCs that exerted a great antitumor effect *via* photothermally enhanced tumor-specific Fenton chemistry.

## Introduction

Reactive oxygen species (ROS) are a series of chemicals originated from complete or partial reduction of oxygen in living organisms. As the main by-products of oxygen metabolism, ROS including H_2_O_2_, ^1^O_2_, ·OH, and O_2_^·-^ play essential roles during the regulation process of various physiological functions, while overproduction of ROS can introduce serious cellular oxidative stress *via* DNA damage, protein denaturation, as well as lipid peroxidation (1–6). Accordingly, numerous efforts have been devoted to using excessive harmful ROS to regulate the intracellular redox status for oncotherapy. Compared with classical approaches of surgery and chemotherapy, current novel ROS-mediated therapeutic strategies with the assistance of exogenous/endogenous light, ultrasound, radiation, and chemical species hold more advantages including non-invasiveness, high selectivity, and negligible side effects (7–13). Although promising, the clinical efficiency of the above strategies in solid tumors is severely restricted by the hypoxic tumor microenvironment and the intratumoral ROS generation efficiency. Thus, it is urgently needed to develop novel therapeutic strategies towards further oxygen-independent ROS-mediated oncotherapy.

Exploitation of the typical Fenton reaction and Fenton-like chemical reaction for oncotherapy has attracted increasing attention in recent years (14–16). Briefly, Fenton reaction containing ferrous ions/ferric ions (Fe^2+^/Fe^3+^) is able to efficiently catalyze the transformation of endogenous H_2_O_2_ into highly toxic ·OH in tumors. Compared with other ROS-mediated antitumor strategies, mild acidity and elevated levels of H_2_O_2_ of tumor microenvironment can significantly endow intratumoral Fenton chemistry with more advantages in therapeutic selectivity and specificity as compared to normal tissues (17–20). However, Fenton reaction alone cannot achieve a satisfactory clinical antitumor efficiency due to the endogenous lake of Fenton catalysts and relatively limited reaction conditions including excess reducing glutathione in tumors. As well known, the amount of Fenton catalysts in tumors is directly related to the generation efficacy of ·OH, and effective delivery of Fenton catalysts to the tumor regions accordingly has been considered as an important feature in a Fenton reaction-mediated oncotherapy (21–23). In addition, recent studies indicate that raising temperature of reaction system can highly increase efficiency of Fenton reaction in some research fields including the treatment of hazardous organic pollutants in water based on the classical thermodynamic molecular collision theory (24–26). Therefore, a strategy to deliver Fenton catalysts and simultaneously increase the temperature of tumor regions can achieve a localized and adaptive Fenton reaction with an enhanced antitumor therapeutic effect.

Thanks to the remarkable breakthroughs in the field of nanotechnology and materials science, a great deal of novel nanomaterials with unique physicochemical properties have been rationally designed and well prepared as next-generation therapeutic reagents for biomedical usages (27–31). As a series of important nanomaterials, magnetic iron oxide nanoparticles have been widely explored in versatile biomedical fields including magnetic resonance (MR) imaging, magnetic hyperthermia, photothermal therapy, chemodynamic therapy, nanozyme-based bioanalysis, magnetic field-assisted targeted drug delivery, and magnetic separation of biomolecules (32–42). Recent studies indicate that magnetic nanoparticles exhibit distinct toxicity under different physiological conditions (43–45). In detail, magnetic nanoparticles can decompose H_2_O_2_ into non-toxic O_2_ and H_2_O under neutral condition with catalase-like activity while they convert H_2_O_2_ into highly toxic ·OH under acidic condition and behave like Fenton nanocatalysts. Significantly, U. S. Food and Drug Administration-approved magnetic nanoparticles named ferumoxytol can inhibit the growth of early mammary cancers and prevent the hepatic metastasis *via* the increase of pro-inflammatory M1 macrophages in tumor regions, which is closely related to the generation of ROS *via* Fenton reaction. In that case, we envision that magnetic nanoparticles can achieve enhanced ROS-mediated oncotherapy with their intrinsic photothermal effect. More importantly, magnetic nanoparticles can efficiently target tumor regions with the assistance of external magnetic field. All these exciting lines of evidence encourage us to using magnetic nanoparticles as new-generation biocompatible antitumor reagents with new therapeutic mechanism and high therapeutic efficiency.

In this study, a novel NIR light-mediated tumor-specific nanoplatform based on magnetic iron oxide nanoclusters (MNCs) was rationally designed and well developed for photothermally enhanced Fenton reaction-assisted oncotherapy. MNCs with excellent magnetic response character could efficiently accumulate into the tumor regions under an external magnet field, favoring further T_2_-weighted MR imaging of tumors and MR imaging-guided combined antitumor therapy. Upon a NIR light irradiation, these MNCs exhibited great photothermal effect, promising their important role as a photothermal therapy (PTT) agent. It is worth noting that heat induced by the PTT of MNCs could accelerate the release of Fe from MNCs and enhance the efficiency of Fenton reaction under H_2_O_2_-enriched acidic tumor microenvironment. Toxicity investigations after intravenous injection of MNCs indicated their overall safety. Taken together, a new magnetic nanoparticle-based therapeutic strategy against solid tumors was developed *via* photothermally enhanced tumor-specific Fenton chemistry, promising for further preclinical or clinical applications.

## Materials and Methods

### Chemicals

FeCl_3_·6H_2_O, urea, polyethylene glycol (PEG, M_w_ = 2000) and sodium citrate were purchased from Aladdin Reagent. Calcein AM and propidium iodide (PI) were achieved from Sigma-Aldrich. Dulbecco’s modified Eagle’s medium (DMEM) and fetal bovine serum (FBS) were obtained from Beyotime Biotechnology. All chemicals were of analytical grade and used directly without any purification. Water used in all experiments was obtained *via* a Milli-Q water system.

### Synthesis of Magnetic Iron Oxide Nanoclusters

Magnetic iron oxide nanoclusters (MCNs) were synthesized *via* a facile one-pot hydrothermal method. Typically, FeCl_3_·6H_2_O (2 mmol), sodium citrate (4 mmol), urea (6 mmol), and PEG (2.0 g) were dissolved in distilled water (40 ml). After the above mixture was dissolved totally, the solution was transferred to a Teflon-lined autoclave (50 ml) and maintained at 200°C for 12 h. After cooling down to room temperature, MCNs were collected by magnetic separation, washed with distilled water and absolute ethanol, and dried under vacuum overnight for further use.

### Photothermal Effect of MNCs

MNCs dispersed in 0.9% NaCl solution with different concentrations were irradiated under an 808-nm laser with a power intensity of 2 W/cm^2^. Solution temperatures were recoded every 30 s by using an infrared thermal camera (FLIR I3). Relative photothermal photos of MNCs after near-infrared irradiation with a period of 5 min were collected at the same time.

### Time-Dependent Fe Release

MNCs were added into various PBS (10 mM, 10 ml) with different pH values under 37°C. Final concentration of MNCs was defined as 1 mg/ml. At each given time interval, solution was collected after magnetic separation of residual MNCs, and the release amount of Fe in each group was quantitatively analyzed *via* ICP-MS. Three independent repeated experiments were done in the abovementioned groups.

### Catalytic Effect of MNCs

A colorimetric method based on the oxidation of TMB was used to explore the Fenton reaction performance of MNCs. Briefly, absorbance changes at 652 nm of TMB at pH 7.4 or 6.5 with or without H_2_O_2_ were measured in the presence or absence of MNCs at 37°C. In order to simulate the heat from the photothermal effect of MNCs, reaction temperature was maintained at 50°C and the temperature effect on the Fenton reaction of MNCs was investigated. Concentrations of MNCs, TMB, and H_2_O_2_ in the above experiments were 50 μg/ml, 1 mM, and 20 mM, respectively. Co-incubation period of above reaction system was defined as 5 min.

### Cell Culture

4T1 mouse breast tumor cell line (4T1 cells) was obtained from the Type Culture Collection Committee of the Chinese Academy of Sciences (Shanghai, China) and was cultured in DMEM containing 10% FBS in an incubator (37°C, 5% CO_2_).

### Cytotoxicity of MNCs

4T1 cells were seeded into a 96-well plate to allow the cellular attachment at first (*n* = 4). Twelve hours later, medium was replaced by medium containing MNCs. After further co-incubation for 24 h, cell viabilities were evaluated by using MTT assay. Moreover, similar experiments were carried out and above cellular suspension was collected for LDH leakage analysis (*n* = 4). For cellular live-dead staining analysis of MNCs, 4T1 cells were seeded into a six-well plate to allow the cellular attachment. Twelve hours later, medium was replaced by medium containing MNCs. After co-incubation for 12 h, medium containing MNCs was removed, live-dead staining was performed, and stained cells were observed under an Olympus BX-51 imaging system.

### Animals

Balb/c mice weighing about 25 g were purchased from Charles River (Beijing, China). The protocol of all the animal studies was approved by the Institutional Animal Care and Use Committee at Jilin University.

### Assays of Hemolysis and Coagulation

Fresh mouse blood samples stabilized by EDTA were obtained from Balb/c mice. After centrifugation at 1,000 rpm, red blood cells were diluted to a quarter of their volumes with 0.9% NaCl solution. Diluted red blood cells (0.2 ml) were mixed with 0.9% NaCl solution (0.8 ml) as negative group, distilled water (0.8 ml) as positive group, and 0.9% NaCl solution containing a series of concentrations of MNCs (0.8 ml) as experimental groups. The above samples were further vortexed and maintained for another 2 h. Then, the absorbance of supernatants at 541 nm was determined *via* a UV-vis spectroscopy (*n* = 4). For coagulation assays, plasma from volunteers was mixed with 0.9% NaCl solution containing MNCs at first. Then, a fully automatic blood coagulation analyzer was used to read out the values of activated partial thromboplastin time (APTT) and prothrombin time (PT) in the presence of MNCs with different co-incubation concentrations (*n* = 4).

### Light-Induced Cytotoxicity of MNCs

4T1 cells were seeded into a 96-well plate and cultured overnight (*n* = 4). Then, medium was replaced by medium containing MNCs. Six hours later, cells were irradiated with an 808-nm laser with different power intensities for 5 min. After another 6 h incubation, cell viabilities were evaluated by using the MTT assay.

### Fenton Reaction-Mediated Cytotoxicity of MNCs

4T1 cells were seeded into a 96-well plate and cultured overnight (*n* = 4). Then, medium was replaced by medium containing a series of concentrations of MNCs and H_2_O_2_ (100 μM) at pH 7.4 or 6.5. After 12 h co-incubation, cell viabilities were evaluated by using MTT assay.

### Combined Antitumor Effect of MNCs *In Vitro*

4T1 cells were seeded into a 96-well plate and cultured overnight (*n* = 4). Cells were divided into eight groups, namely, control, NIR, H_2_O_2_, H_2_O_2_+NIR, MNCs, MNCs+H_2_O_2_, MNCs+NIR, and MNCs+H_2_O_2_+NIR. MNCs with a concentration of 100 μg/ml, H_2_O_2_ with a concentration of 100 μM, pH value of 6.5, an 808-nm laser with a power intensity of 2 W/cm^2^, and an irradiation period of 5 min were used throughout our current experimental design. Twelve hours after the above various treatments, cell viabilities of various groups were evaluated by using the MTT assay.

### Live/Dead Staining

4T1 cells were seeded into a six-well plate and cultured overnight. Then, medium was replaced by fresh medium with different components. Detailed treatments were similar to those of combined antitumor effect of MNCs *in vitro*. Acidified medium was used throughout all groups except the control group. After the above treatments, cells were stained with calcein AM and PI, and fluorescence microscopy images were collected on an Olympus BX-51 imaging system. In addition, trypan blue solution (0.4%) was used to distinguish live/dead cells under an optical microscope.

### Cellular ROS Detection

4T1 cells were seeded into a six-well plate and cultured overnight. Then, medium was replaced by fresh medium with different components. Detailed treatments were similar to those of combined antitumor effect of MNCs *in vitro*. Acidified medium was used throughout all groups except the control group. After the above treatments, cells were washed with cool 0.9% NaCl solution. After the co-incubation with DCFH-DA for another 20 min, cells were observed under an Olympus BX-51 imaging system.

### Preparation of 4T1 Tumor-Bearing Mice

4T1 tumor xenografts were implanted into the axillaries of Balb/c mice by subcutaneous injection of 10^6^ 4T1 cells. When tumor volumes reached about 100 mm^3^, MR imaging, thermal imaging, and antitumor effect of MNCs were explored in detail.

### MR Imaging

MR imaging was carried out on a 1.5-T clinical MR imaging instrument (Siemens Medical System). A series of concentrations of MNCs fixed in low concentration of agarose gel were mixed with water at first and prepared for the following *in vitro* T_2_-weighted MR imaging. For *in vivo* T_2_-weighted MR imaging, 0.9% NaCl solution containing MNCs (1 mg/ml, 1 ml) was intravenously injected into tumor-bearing mice under the assistance of magnet. Mice were imaged before intravenous administration and 5 min after injection of MNCs under the above MR imaging system.

### Thermal Imaging

Firstly, 0.9% NaCl solution containing MNCs (1 mg/ml, 1 ml) was intravenously injected into tumor-bearing mice under the assistance of magnet. Secondly, tumors were irradiated by an 808-nm laser with a power density of 2 W/cm^2^ for 5 min to obtain thermal imaging. During the irradiation, the spot size of laser beam was well adjusted to cover the whole region of tumors. Tumor-bearing mice without intravenous administration of MNCs was selected as the control group to highlight the photothermal effect of MNCs *in vivo*. Both groups were imaged by using an infrared thermal camera. Meanwhile, temperature changes around tumors were recorded.

### Antitumor Effect *In Vivo*

When tumor volumes reached about 100 mm^3^, mice were randomly divided into four groups including control, MNCs, NIR, and MNCs+NIR (*n* = 4). 0.9% NaCl solution containing MNCs (1 mg/ml, 1 ml) was intravenously injected under the assistance of magnet in the groups of MNCs and MNCs+NIR. 1 h after the injection of MNCs, tumors were irradiated with an 808-nm laser (2 W/cm^2^, 5 min) in the group of MNCs+NIR. To achieve a great antitumor effect, the above irradiation was conducted every other day for three treatments. Similar irradiation treatment was also carried out in the group of NIR. Tumor sizes of the above four groups were measured every day during the whole experimental period. Tumor volumes were calculated *via* the following formula: volume = (tumor length) × (tumor width)^2^/2. Two weeks after the first irradiation treatment, mice were sacrificed, tumors were excised and weighed, and tumor sections were examined after hematoxylin and eosin (H&E) staining.

### Biosafety of MNCs

For biosafety evaluation of MNCs, mice with similar body weight were randomly divided into two groups, which were defined as control and test (*n* = 4). Mice in the test group were intravenously injected of high dosages of MNCs (5 mg pre mouse weighted about 25 g). Mice in the control group were administered of 0.9% NaCl solution with the same volume. Body weight measurements of the above mice were recorded for a month. Then, blood of mice in the above groups was collected for further assays of blood biochemical and inflammatory cytokines. Last but not least, mice in the above two groups were sacrificed, and main organs including heart, liver, spleen, lung, and kidney were harvested for further H&E staining.

### Statistical Analysis

All data were expressed as mean ± standard deviation and carried out at least three times. A *p*-value < 0.05 was considered as statistical significances. Statistical analysis was performed *via* one-way analysis of variance together with a *post hoc* LSD test.

## Results and Discussion

**Figure 1:**
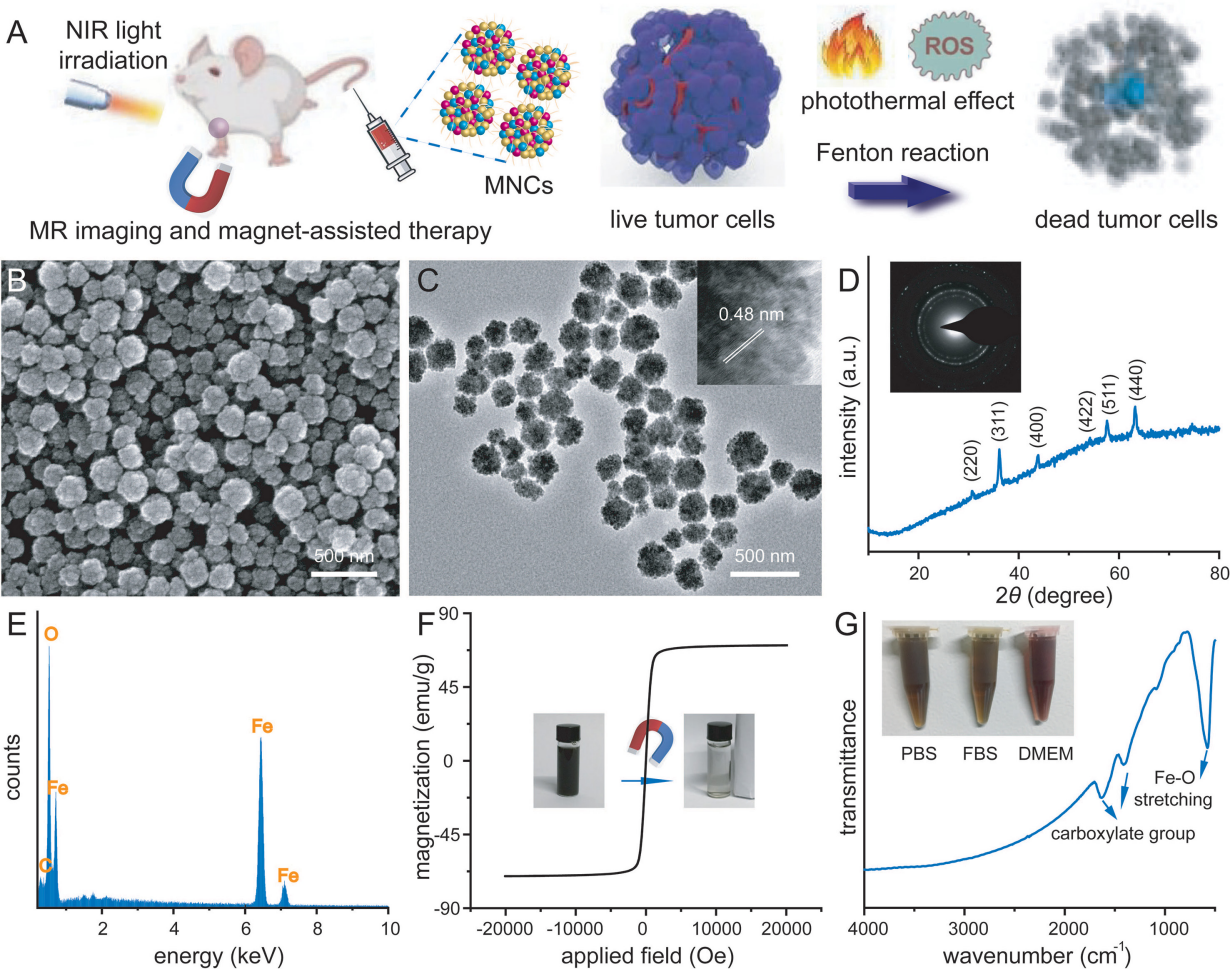
Schematic illustration of tumor-targeting therapy *via* a PTT-enhanced Fenton reaction and relative antitumor mechanism **(A)**. SEM image **(B)**, TEM image **(C)**, wide-angle XRD pattern **(D)**, EDS analysis **(E)**, room temperature magnetic hysteresis loop **(F)**, and FT-IR spectrum **(G)** of well-prepared magnetic nanoclusters (MNCs). Inset of **(C)**: HR-TEM image of MNCs. Inset of D: SEAD image of MNCs. Inset of **(F)**: photo of magnetic separation process. Inset of **(G)**: photos of MNCs dispersed in different physiological solutions.

**Figure 1A** schematically illustrated our rational strategy of NIR light-mediated tumor-specific Fenton reaction-assisted oncotherapy and relative proposed antitumor mechanism of magnetic iron oxide nanoclusters (MNCs). With the help of external magnetic field, MNCs could efficiently target and accumulate into 4T1 tumor sites after intravenous injection. Upon multiple NIR irradiation, MNCs could elevate the temperature of tumor sites, resulting in an effective PTT against solid tumors. During the whole process of PTT, the heat could accelerate the release of Fe from MNCs with the assistance of acid tumor microenvironment, improved the conversion of endogenous H_2_O_2_ into ·OH, and achieved a PTT-enhanced catalytic cancer therapy. Typically, MNCs were well synthesized *via* a facile one-pot hydrothermal method according to previous studies with some modification (40). Images achieved by scanning electron microscopy (SEM) and transmission electron microscopy (TEM) demonstrated the uniform and non-aggregated characteristic of MNCs, which held an average diameter of 140 nm (**Figures 1B, C**). Wide-angle x-ray diffraction (XRD) pattern of MNCs showed a pure-phase cubic structure, promising their well-defined magnetite phase (**Figure 1D**). High-resolution TEM image revealed that MNCs were composed of many smaller particles (inset of **Figure 1C**). Interlayer distance could be calculated as 0.48 nm, which matched well with the separation between (111) lattice planes. A selective area electronic diffraction (SAED) pattern exhibited polycrystalline-like diffraction rings and further supported the polycrystalline nature of MNCs (inset of **Figure 1D**). Energy-dispersive spectroscopy (EDS) analysis confirmed that MNCs were composed of Fe, O, and C elements (**Figure 1E**). The absence of a hysteresis loop based on field-dependent magnetization measurement demonstrated the superparamagnetic nature of MNCs with a relatively high saturation magnetization value of 70.43 emu/g at room temperature (**Figure 1F**). In addition, the photo inset of **Figure 1F** revealed a direct magnetic response of MNCs in water towards magnet. C-H vibrating and carboxylate vibrating in FT-IR spectra indicated the presence of PEG molecule and citrate capped on MNCs, which endowed their great dispersion in various physiological solution including PBS, FBS, and DMEM (**Figure 1G**). Moreover, the addition of PEG molecules and citrate in the typical synthesis implied the presence of negative charge density on the surface of MNCs according to their negative *Z*-potential value. The above results thus promised that our MNCs prepared in aqueous solution instead of organic solvents exhibited more advantages in further biomedical applications.

**Figure 2:**
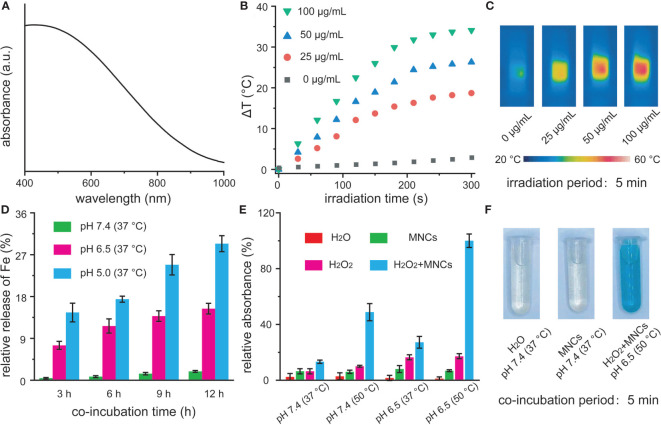
UV-vis-NIR absorption spectra of MNCs in water **(A)**. Temperature change **(B)** and relative near-infrared photos **(C)** of MNCs under an 808-nm laser irradiation with a power intensity of 2 W/cm^2^. Time-dependent Fe release percentages from MNCs with a concentration of 1 mg/ml at different pH values **(D)**. Relative absorbance of TMB at 652 nm **(E)** and corresponding photos **(F)** after various treatments. The concentrations of MNCs, TMB, and H_2_O_2_ used in **(E, F)** were 50 μg/ml, 1 mM, and 20 mM, respectively.

UV-vis-NIR spectra demonstrated that MNCs in water exhibited a broad absorption in the NIR region, which inspired us to explore their photothermal performance (**Figure 2A**). To assess the photothermal properties of MNCs, temperature changes of dispersion containing MNCs with different concentrations were recorded under continuous NIR irradiation (35, 36). As shown in **Figure 2B**, obvious concentration- and irradiation period-dependent temperature increases could be clearly detected, which were then visually confirmed *via* relative NIR thermal imaging photos (**Figure 2C**). In detail, the temperature of a dispersion containing MNCs with a concentration of 100 μg/ml increased by 34.1°C within 5 min of NIR irradiation while the temperature of water increased only 2.5°C. All these results suggested that MNCs could efficiently convert NIR irradiation energy into heat energy. As another vital feature of NIR light-mediated Fenton antitumor therapy, we further explored the release performance of Fe from MNCs under different stimulation conditions. In general, tumor microenvironment was featured with regional hypoxia, low pH, and nutritional deprivation. Moreover, lysosome produced by the rupture of Golgi apparatus usually contained many acid enzymes, and the pH value of lysosome was about 5.0. Therefore, pH 6.5 and pH 5.0 were used to simulate the acid conditions of tumor microenvironment and lysosome, respectively. As shown in **Figure 2D**, a clear time- and pH-dependent Fe release behavior could be found in MNCs with different conditions. After 12 h of co-incubation, 28% Fe release could be detected at pH 5.0 and 17% Fe release occurred at pH 6.5 while less than 3% Fe release was found at pH 7.4. These results thus promised that MNC-based Fenton antitumor therapy could efficiently work in tumor sites instead of other normal tissues or organs. In order to simulate the heat from photothermal effect of MNCs, we further explored reaction temperature effect on the Fenton reaction of MNCs. Based on our experimental design, TMB was used to study the catalytic performance of MNCs by monitoring the generation of ·OH. As shown in **Figure 2E**, upon the addition of MNCs, solution containing TMB and H_2_O_2_ held a temperature- or pH value-dependent manner. Significantly, high temperature or low pH value could lead to obvious absorbance increase at 652 nm. More importantly, maximum absorbance reached only when the experimental condition was defined as low pH value of 6.5 and high temperature of 50°C. Generally, only when the localized photothermal temperature was over 43°C could cellular apoptosis and necrosis be well achieved. Meanwhile, low temperature-induced photothermal effect could be easily resisted by the intracellular regulation, such as heat shock protein-assisted physiological process. Similar to a very recent study, 50°C was therefore selected and used as the typical experimental temperature in our study (24). According to the above findings, heat and low pH value could extremely improve the efficiency of Fenton reaction catalyzed by MNCs, which were also confirmed by the photos of oxTMB colorimetry experiments (**Figure 2F**).

**Figure 3:**
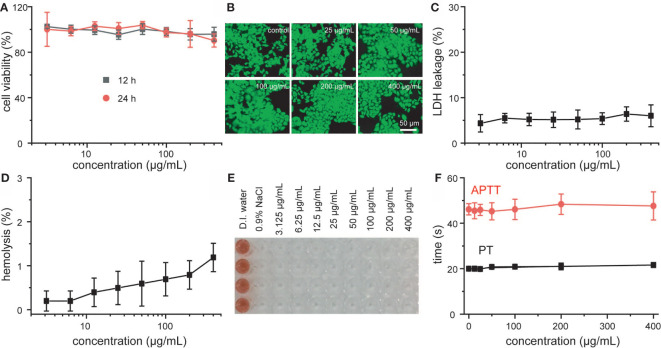
Viability **(A)**, live-dead staining images **(B)**, and LDH leakage **(C)** of 4T1 cells treated with MNCs. Hemolysis assay **(D)**, photo of hemolysis **(E)**, and blood coagulation assay **(F)** after co-incubation with MNCs.

Prior to the use of MNCs for further biomedical usages, their cytotoxicity and blood compatibility were well explored at first. According to the results of MTT assay and live/dead staining shown in **Figures 3A, B**, all the viabilities of 4T1 cells were not hindered after the co-incubation with different concentrations of MNCs, and cellular morphology was not affected by MNCs with the same experimental concentrations, which indicated that MNCs possessed negligible cytotoxicity. Moreover, the results based on lactose dehydrogenase (LDH) leakage revealed that nearly no LDH could be detected even under the maximum co-incubation concentration, promising the integrity of cell membrane in the presence of MNCs (**Figure 3C**). As an essential method towards the exploration of interaction between any newly developed nanomaterial and blood components, hemolytic assay in the presence of MNCs was further investigated. Based on both qualitative and quantitative results shown in **Figures 3D, E** and **Table S1**, MNCs were not even able to cause slight injury of red blood cells, and all the hemolysis percentages were lower than 2%. In addition, activated partial thromboplastin time (APTT) and prothrombin time (PT) were used to explore the blood coagulation of MNCs. As expected, no obvious differences in APTT and PT could be found between the samples treated with a series of concentrations of MNCs and the reference one, thus demonstrating the negligible effect of MNCs on blood coagulation time (**Figure 3F**). All of these exciting results indicated the negligible cytotoxicity and high blood compatibility of MNCs.

**Figure 4:**
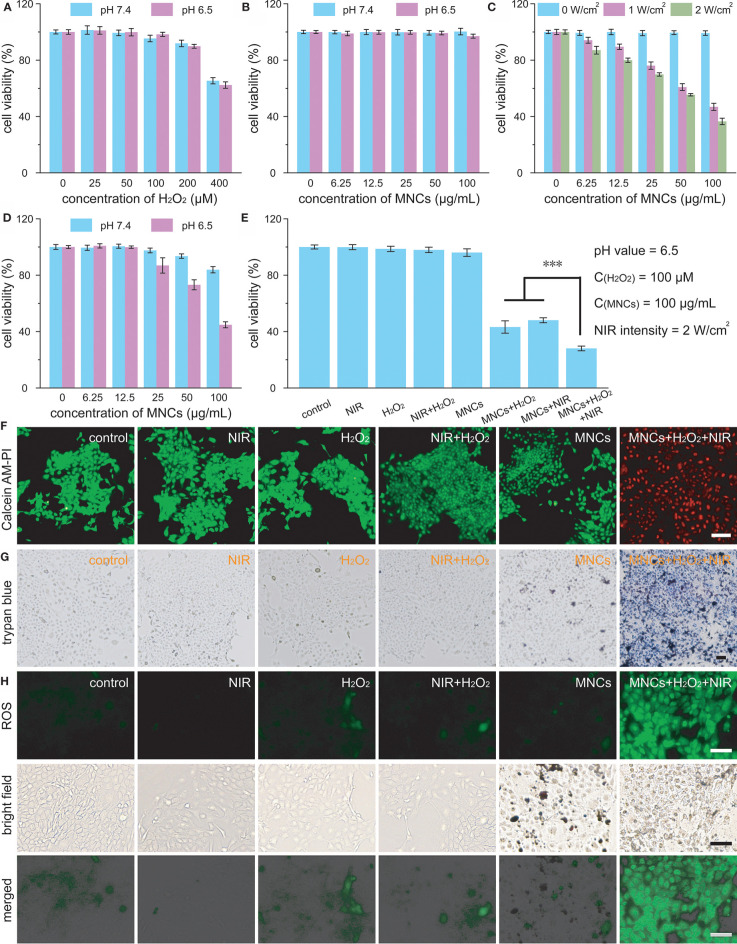
Viabilities of 4T1 cells treated with H_2_O_2_ **(A)** and MNCs **(B)** under different pH values. Cellular viabilities under laser irradiation in the presence of MNCs **(C)**. Cellular viabilities under different pH values in the presence H_2_O_2_ and MNCs **(D)**. The concentration of H_2_O_2_ in D was 100 μM. Combined *in vitro* antitumor effect of MNCs with laser irradiation and H_2_O_2_ under acidic pH value **(E)**. Inset of E: detailed experimental conditions. Live-dead staining images **(F)**, trypan blue staining images **(G)**, and ROS staining images **(H)** of 4T1 cells after various treatments. Scale bars are equal to 100 μm. Asterisks indicated statistical significances (*p < 0.05, **p < 0.01, ***p < 0.001).

Encouraged by the excellent properties of MNCs so far, we further investigated their antitumor effect *in vitro*. Cytotoxicity of 4T1 cells against H_2_O_2_ under different pH values was explored at first in order to confirm the experimental concentration of H_2_O_2_ in cell culture. As shown in **Figure 4A**, 100 μM was considered as a reasonable concentration of H_2_O_2_ for further cell culture because 200 μM or 400 μM of H_2_O_2_ could induce more or less death of 4T1 cells upon different co-incubation pH values including pH 7.4 and pH 6.5 (23–25). Moreover, no apparent cytotoxicity could be detected even at a high concentration of MNCs up to 100 μg/ml, both at pH 7.4 and pH 6.5, further demonstrating their high biocompatibility (**Figure 4B**). With the assistance of an 808-nm laser irradiation, viabilities of 4T1 cells significantly decreased along with the increasing of the concentrations of MNCs or laser intensities (**Figure 4C**). For instance, fewer than 40% of 4T1 cells survived when treated with MNCs at 100 μg/ml and a 2 W/cm^2^ laser irradiation, indicating the excellent PTT effect of MNCs *in vitro.* We then evaluated the Fenton reaction-mediated antitumor effects of MNCs. As shown in **Figure 4D**, MNCs exhibited a slight cytotoxicity when incubated at pH 7.4 with H_2_O_2_, while an obvious concentration-dependent antitumor effect was found at pH 6.5 with H_2_O_2_. In detail, viability of 4T1 cells decreased to their 40% in the presence of 100 μg/ml of MNCs at pH 6.5 with 100 μM of H_2_O_2_. In the following experiments, we further explored the NIR laser-assisted combined antitumor effect of MNCs in the presence of H_2_O_2_ at relatively low pH value, which was used to simulate tumor microenvironment. Detailed experimental information was illustrated in **Figure 4E**. No more than 60% of 4T1 cells were killed in the groups of MNCs+H_2_O_2_ and MNCs+NIR; however, more than three-quarters of 4T1 cells were killed in the group of MNCs+H_2_O_2_+NIR, which exhibited a clearly enhanced antitumor effect than PTT alone or Fenton reaction alone. Live-dead staining images and trypan blue staining images further demonstrated the combined antitumor effects of MNCs *via* both PTT and Fenton reaction (**Figures 4F, G**). No dead cells could be detected in the groups of control, NIR, H_2_O_2_, NIR+H_2_O_2_, and MNCs. However, nearly all the 4T1 cells were damaged or killed in the group of MNCs+H_2_O_2_+NIR. The generation of ROS in 4T1 cells by Fenton reaction was then detected with the help of DCFH-DA. As shown in **Figure 4H**, green fluorescence could be easily found in the groups with the addition of H_2_O_2_. Compared with that of the group of H_2_O_2_ or the group of NIR+H_2_O_2_, much stronger fluorescence could be detected in the group of MNCs+H_2_O_2_+NIR, which could be ascribed to the effect of photothermally enhanced Fenton reaction. All these exciting results thus demonstrated the excellent combined antitumor effects of MNCs under simulated tumor microenvironment.

**Figure 5:**
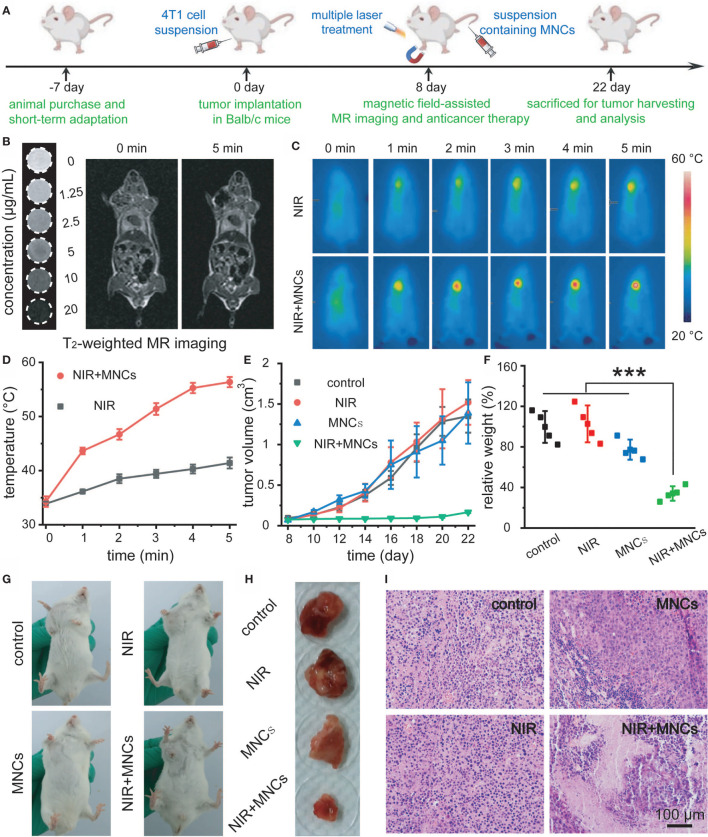
Schematic illustration of the timeline for NIR light-mediated tumor-specific Fenton reaction-assisted oncotherapy **(A)**. T_2_-weighted MR image of MNCs in aqueous solution and *in vivo* magnet-assisted MR imaging before and 5 min after intravenous administration of MNCs **(B)**. Thermal images of tumor-bearing mice **(C)** and relative temperature profiles of tumor sites in mice **(D)** under different treatments. Tumor growth curves **(E)** and relative tumor weights at the end of the whole therapy period **(F)** for the indicated treatments. Photos of tumors in mice **(G)** and relative tumor tissues collected from mice **(H)** at the end of the whole therapy period upon different treatments. H&E staining of tumor tissues after various treatments **(I)**. Asterisks indicated statistical significances (*p < 0.05, **p < 0.01, ***p < 0.001).

**Figure 5A** schematically illustrated the detailed timeline for NIR light-mediated tumor-specific Fenton reaction-assisted oncotherapy. When tumor volumes grew to 100 mm^3^, 0.9% NaCl solution containing MNCs was intravenously injected with the assistance of an external magnetic field around the tumors. Then, an 808-nm laser with a power density of 2 W/cm^2^ was used to perform irradiation for the following tumor thermal imaging and tumor ablation experiment. Moreover, magnetic field-assisted MR imaging was carried out at the same time owing to the excellent magnetic property of MNCs. As shown in **Figure 5B**, all the tubes containing MNCs appeared darker and darker according to T_2_-weighted MR images along with the increasing of concentrations of MNCs while the tube without the addition of MNCs remained bright. *In vivo* T_2_-weighted images before and after administration of MNCs demonstrated that our well-designed MNCs could efficiently accumulate into tumor upon the help of magnetic field even within a very short response period of 5 min. In detail, a significant darkening effect in the tumor region of 4T1 tumor-bearing mouse could be easily detected as compared with the image recorded before administration of MNCs. These results thus indicated the feasibility of MNCs as MR imaging-guided antitumor theranostic reagents. NIR light-induced temperature changes *in vivo* by MNCs were then explored with the assistance of a thermal imaging camera. As shown in **Figures 5C, D**, the temperature of tumor region administered with MNCs could easily increase from mouse body temperature to about 55°C during the whole NIR light irradiation, while other regions, which were not exposed to the NIR light, exhibited no temperature changes. Additionally, a no more than 5°C increase occurred around the tumor region without the injection of MNCs. Before the evaluation of antitumor effects of MNCs *in vivo*, 4T1 tumor-bearing mice were randomly divided into four groups at first, which were then defined as control, MNCs, NIR, and MNCs+NIR. During the whole treatment period of 2 weeks, tumor volumes were monitored to assess the therapeutic effects of above groups. As shown in **Figure 5E**, groups of control, NIR, and MNCs exhibited relatively rapid tumor growth while nearly total tumor suppression could be easily found in the group of MNCs+NIR. At the end of the whole antitumor treatment period, mice were sacrificed, and tumors were excised and weighted. As shown in **Figures 5F**–**H**, average tumor weights in the above four groups were consistent with our tumor volume measurements. However, tumor weights in the group of MNCs revealed somewhat lighter than those in the groups of control and NIR because of the appearance of partial tumor cavity after tumor ablation, indicating the effects of Fenton reaction-mediated antitumor therapy. Significantly, photothermal effect of MNCs could extremely enhance the effect of Fenton reaction based on our current study. **Figure 5I** provided the H&E staining of tumor sections collected from the mice at the end of antitumor treatment. As expected, no obvious tumor cell damage was found in the groups of control and NIR. However, most tumor cells in the group of MNCs+NIR were killed while some cell damage was detected in tumors from mice treated with MNCs, which re-confirmed the superior antitumor effect of MNCs based on the rationally combined PTT and Fenton reaction.

**Figure 6:**
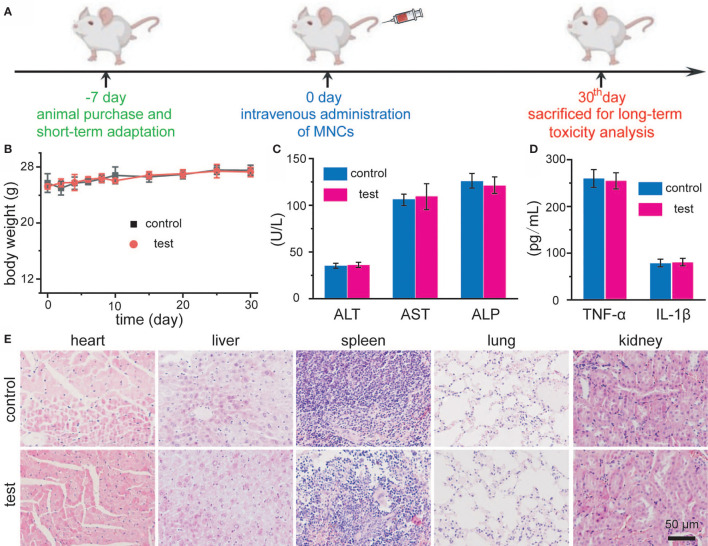
Schematic illustration of *in vivo* toxicity evaluation of MNCs **(A)**. Body-weight changes of Balb/c mice after intravenous injection of MNCs **(B)**. Serum levels of ALT, AST, and ALP in mice **(C)**. Serum levels of TNF-α and IL-β in mice **(D)**. H&E staining images of mouse main exposed organs after different treatments **(E)**.

To determine whether MNCs could induce any harmful effect after systemic administration, we then explored the long-term toxicity of MNCs. The timeline of our experimental design is illustrated in **Figure 6A**. Firstly, mice adapt to diet about 1 week before the initiation of experimentation. Secondly, MNCs with a relative high dosage dispersed in 0.9% NaCl solution were intravenously injected. As shown in **Figure 6B**, no differences in body weight could be detected between the control group and the test group during the whole experimental period of a month. In addition, mice in both groups did not exhibit any behavior abnormality. A month after the intravenous injection of MNCs, assays of blood biochemicals and inflammatory cytokines were used to provide more quantitative toxicity evaluation of these MNCs. Results shown in **Figure 6C** demonstrated that MNCs did not alter the hepatic function indexes, such as alanine aminotransferase (ALT), alkaline phosphatasein (ALP), and aspartate aminotransferase (AST). Serum levels of inflammatory cytokines including TNF-α and IL-β in mice exhibited negligible differences at the same time (**Figure 6D**). H&E histology analysis of main exposed organs a month after the injection of MNCs was used to indicate the potential signs of inflammation and injuries. Compared with those of the control group, no obvious inflammation and injuries were observed in the test group (**Figure 6E**). All these results thus supported the extremely low systemic toxicity of MNCs and the high feasibility of our current therapeutic strategy.

## Conclusion

In summary, a novel NIR light-mediated tumor-specific nanoplatform based on efficient MNCs has been well developed for photothermally enhanced Fenton reaction-assisted oncotherapy. These MNCs were synthesized *via* a green hydrothermal method without any organic solvent. By virtue of their superparamagnetic nature, these MNCs can act as a T_2_-weighted MR imaging-guided antitumor theranostic reagent. Meanwhile, relatively high saturation magnetization value endows these MNCs with the capacity for tumor targeting with the help of an external magnet field. Because of their high photothermal effect, MNCs can serve as an efficient PTT agent for antitumor therapy. Significantly, heat produced by the PTT effect can extremely accelerate the release of Fe from MNCs and achieve an enhanced Fenton reaction under H_2_O_2_-enriched acidic tumor microenvironment for oncotherapy. Both *in vitro* and *in vivo* results confirm the superior antitumor effect of MNCs based on combined PTT and Fenton reaction, promising the high feasibility of our current therapeutic strategy. Last but not least, long-term toxicity exploration of MNCs after intravenous administration suggests their low systemic toxicity and high biocompatibility. Accordingly, our present approach not only develops a new concept of photothermally enhanced Fenton chemistry based on magnetic nanomaterials, but also provides a meaningful insight for further nanoplatform-assisted combined tumor therapy.

## Data Availability Statement

The original contributions presented in the study are included in the article/**Supplementary Material**. Further inquiries can be directed to the corresponding author.

## Ethics Statement

The animal study was reviewed and approved by the Institutional Animal Care and Use Committee at Jilin University.

## Author Contributions

SQ and JX contributed equally to this work. SQ, JX, and CZ conceived and designed the experiments and wrote the manuscript. SQ, JX, EJ, NR, and YD performed the experiments. All authors contributed to the article and approved the submitted version.

## Funding

Financial support was provided by the Jilin Provincial Health Special Project (2018SCZ015) and the National Natural Science Foundation of China (81900521).

## Conflict of Interest

The authors declare that the research was conducted in the absence of any commercial or financial relationships that could be construed as a potential conflict of interest.

## Publisher’s Note

All claims expressed in this article are solely those of the authors and do not necessarily represent those of their affiliated organizations, or those of the publisher, the editors and the reviewers. Any product that may be evaluated in this article, or claim that may be made by its manufacturer, is not guaranteed or endorsed by the publisher.
