# Supplementary material for: Achieving NIR Light-Mediated Tumor-Specific Fenton Reaction-Assisted Oncotherapy by Using Magnetic Nanoclusters
Source: Front Oncol. 2021 Oct 25;11:777295. doi: 10.3389/fonc.2021.777295 (PMC8573244; doi:10.3389/fonc.2021.777295)
Supplement: Supplementary file 1 [file DataSheet_1.pdf]

Supporting Information for

Achieving NIR Light-Mediated Tumor-Specific Fenton Reaction-Assisted Oncotherapy by Using Magnetic Nanoclusters

Shaoyou Qin,[†] Jinru Xue,[‡] Erna Jia,[†] Na Ren,[‡] Yongqiang Dong,[§] and Changyu Zhou [,†]*

[†] Department of Gastroenterology and Hepatology, China-Japan Union Hospital of Jilin University, Jilin University, Changchun 130033, P.R. China

[‡] Department of Thoracic Surgery, China-Japan Union Hospital of Jilin University, Jilin University, Changchun 130033, P.R. China

[§] Department of Thyroid Surgery, First Affiliated Hospital of Zhengzhou University, Zhengzhou 450052, P.R. China.

E-mail: cyzhou@jlu.edu.cn

Table S1. Mean value of hemolysis percentages after co-incubation with MNCs.

concentrations (μg/mL)	average value of hemolysis (%)
0.9% NaCl	0
D.I. water	100
3.125	0.198
6.25	0.198
12.5	0.396
25	0.495
50	0.594
100	0.693
200	0.792
400	1.188
